# Analysis of Orthopaedic In-Training Examination Trauma Questions: 2017 to 2021

**DOI:** 10.5435/JAAOSGlobal-D-22-00180

**Published:** 2023-03-13

**Authors:** Lilah Fones, Daryl C. Osbahr, Daniel E. Davis, Andrew M. Star, Atif K. Ahmed, Arjun Saxena

**Affiliations:** From the Rothman Orthopaedic Institute at Thomas Jefferson University, Philadelphia, PA.

## Abstract

The Orthopaedic In-Training Examination (OITE) is a multiple-choice examination developed by the American Academy of Orthopaedic Surgeons annually since 1963 to assess orthopaedic residents' knowledge. This study's purpose is to analyze the 2017 to 2021 OITE trauma questions to aid orthopaedic residents preparing for the examination.

**Methods:**

The 2017 to 2021 OITEs on American Academy of Orthopaedic Surgeons' ResStudy were retrospectively reviewed to identify trauma questions. Question topic, references, and images were analyzed. Two independent reviewers classified each question by taxonomy.

**Results:**

Trauma represented 16.6% (204/1,229) of OITE questions. Forty-nine percent of trauma questions included images (100/204), 87.0% (87/100) of which contained radiographs. Each question averaged 2.4 references, of which 94.9% were peer-reviewed articles and 46.8% were published within 5 years of the respective OITE. The most common taxonomic classification was T1 (46.1%), followed by T3 (37.7%) and T2 (16.2%).

**Discussion:**

Trauma represents a notable portion of the OITE. Prior OITE trauma analyses were published greater than 10 years ago. Since then, there has been an increase in questions with images and requiring higher cognitive processing. The *Journal of Orthopaedic Trauma* (24.7%), *Journal of the American Academy of Orthopaedic Surgeons* (10.1%), and *Journal of Bone and Joint Surgery, American Volume* (9.3%) remain the most cited sources.

The Orthopaedic In-Training Examination (OITE) is a multiple-choice examination developed by the American Academy of Orthopaedic Surgeons (AAOS) annually since 1963 to assess orthopaedic residents' knowledge during training.^1^ Currently, the examination is administered to over 4,000 residents annually.^2^ The AAOS reports that examination content is divided into 11 key topics: foot and ankle, hand, hip and knee, orthopaedic basic sciences, orthopaedic oncology, pediatrics, practice management, shoulder and elbow, spine, sports medicine, and trauma. Trauma is reported to represent approximately 15% of the examination content annually.^3^

The OITE is an important examination for both residents and program directors. For residents, the OITE scores, particularly in the last year of postgraduate training, correlate with the American Board of Orthopaedic Surgery part I scores.^4-6^ For program directors, the OITE is the only national test to objectively assess a resident's orthopaedic knowledge both as a benchmark on the efficacy of the residency education curriculum and on the progress of their individual residents.^6^ Given the importance of the examination, most residents are motivated to perform well, and most rely on an online learning platform, most commonly ResStudy and Orthobullets, to aid in preparation for the OITE. Only a minority of residents use journal reading as their primary learning material.^7^

Prior analyses of the trauma section of the OITE were published greater than 10 years ago and studied the OITE between 2004 and 2009.^8-10^ These prior studies analyze questions preceding the conversion to a computer-based examination in 2009, which expanded the opportunity to include more advanced imaging and videos as part of the examination and thus could have influenced the examination content and the types of images included.^9^ Recently, multiple studies have published an updated analysis of OITE questions in other subspecialties, including pediatrics,^11^ oncology,^12^ hand,^13^ hip and knee reconstruction,^14^ basic science,^15^ spine,^16^ and sports.^17^ To our knowledge, no update on the analysis of the OITE trauma section has been published.

The primary purpose of this study is to determine the representation of trauma questions on the OITE between 2017 and 2021 and to analyze these questions for topic, references, imaging, and question taxonomy. Study results will help inform current orthopaedic residents as they prepare for the annual examination and residency program directors as they develop their program's educational curriculum.

## Methods

The 2017 to 2021 OITE questions published on AAOS's ResStudy online platform were retrospectively reviewed. Questions testing concepts related to musculoskeletal trauma were compiled for analysis. Questions related to traumatic injuries that could otherwise be categorized as spine or hand were excluded. This exclusion criterion aims for the questions analyzed to best reflect the type of injuries that are typically managed by a trauma-focused general orthopaedic surgeon and test orthopaedic trauma principles. By contrast, traumatic injuries to the hand and spine are typically treated by their respective subspecialists, and the authors believe that questions related to these topics are better analyzed in studies of the hand and spine domains of the OITE, respectively. For each question, all associated references, figures, and images were compiled. The percentage of trauma questions for each OITE examination was calculated. The topic of each question was categorized as shoulder and elbow, hip and femur, knee and leg, foot and ankle, pelvis and acetabulum, basic science, and general trauma principles. The number of references for each question was recorded. References were classified by publication year. The year of publication was compared with the year of the OITE, and the time elapsed between the reference publication and the OITE was recorded. For questions with figures, both the number of images included for each question and the imaging modality (radiograph, CT, magnetic resonance imaging, clinical photograph, or diagram) were identified. Two independent reviewers classified each question by taxonomy, as previously defined by Buckwalter et al.^18^ Taxonomy I questions (T1) assess information recognition and recall. Taxonomy II questions (T2) test data comprehension and interpretation through diagnosis or image interpretation. Taxonomy III questions (T3) test knowledge application for problem solving, such as multistep management or decision making. Chi-squared tests were used to compare categorical data. *P* < 0.05 was considered statistically significant.

## Results

Trauma questions represented 16.6% (204/1,229) of OITE questions between 2017 and 2021, ranging from 14.0 to 20.9% annually. Topics tested include shoulder and elbow (19.1%), hip and femur (18.6%), knee and leg (12.7%), foot and ankle (14.7%), pelvis and acetabulum (14.2%), basic science (10.8%), and general trauma principles (9.8%), as summarized in Table 1.

**Table 1:** Total Number of Questions and Question Topic in the Trauma Section of Orthopaedic In-Training Examination

	2017	2018	2019	2020	2021	Total
Total questions	271	270	260	215	213	1229
Trauma questions	38	48	41	45	32	204 (16.6%)
Shoulder/elbow	5	12	15	4	3	39 (19.1%)
Hip/femur	5	10	5	11	7	38 (18.6%)
Knee/leg	7	5	4	4	6	26 (12.7%)
Foot/ankle	6	3	3	11	7	30 (14.7%)
Pelvis/acetabulum	5	10	6	5	3	29 (14.2%)
Basic science	6	5	1	7	3	22 (10.8%)
General trauma	4	3	7	3	3	20 (9.8%)

Images were included in 49.0% (100/204) of questions and 87.0% (87/100) of these contained radiographs, 67.0% of which included radiographs alone, and 20.0% included radiographs in combination with other imaging modalities. Other imaging modalities included were CT (21.0%), clinical images (8.0%), and diagrams (4.0%). Table 2 summarizes the representation of images in each OITE.

**Table 2:** Summary of Trauma Questions Containing Imaging

Imaging Modality	2017	2018	2019	2020	2021	Total	Percentage of Questions
No images	21	27	19	20	17	104	51.0% (104/204)
Images	17	21	22	25	15	100	49.0% (100/204)
Radiograph	8	19	17	15	8	67	67.0% (67/100)
CT	2	1	0	1	1	5	5.0% (5/100)
Radiograph and CT	4	0	3	7	2	16	16.0% (16/100)
Clinical image	0	1	1	1	1	4	4.0% (4/100)
Radiographs and clinical image	3	0	1	0	0	4	4.0% (4/100)
Diagram	0	0	0	1	3	4	4.0% (4/100)

On average, there are 2.4 references per question (ranging from 2 to 6). Peer-reviewed journal articles represent 94.9% of all references (ranging from 92.0% to 97.2%), whereas the remaining 5.1% of references were composed of textbooks, websites, and instructional course lectures. The three most common journals cited were the *Journal of Orthopaedic Trauma* (24.7%), the *Journal of the American Academy of Orthopaedic Surgeons* (10.1%), and the *Journal of Bone and Joint Surgery, American Volume* (9.3%). These top three journals account for 44.1% of references. Table 3 summarizes each journal accounting for at least 1% of all references. Nearly half (46.8%) of references were published within 5 years of the respective OITE. Table 4 summarizes the publication year of each reference in relation to the OITE it was cited on.

**Table 3:** Journal Articles Representing 1% or More of All References

Journal	2017	2018	2019	2020	2021	Total	Percentage of Questions
*Journal of Orthopaedic Trauma*	24	24	25	29	20	122	24.7
*Journal of the American Academy of Orthopaedic Surgeons*	2	20	15	7	6	50	10.1
*Journal of Bone and Joint Surgery, American Volume*	7	9	8	16	6	46	9.3
*Injury*	5	9	7	5	7	33	6.7
*Clinical Orthopaedics and Related Research*	3	2	5	6	1	17	3.4
*Foot & Ankle International*	1	2	6	2	2	13	2.6
*Journal of Bone and Joint Surgery, British Volume*	1	4	3	4	1	13	2.6
*Journal of Shoulder and Elbow Surgery*	1	2	5	3	2	13	2.6
*The Bone & Joint Journal*	0	4	0	3	2	9	1.8
*Journal of Trauma and Acute Care Surgery*	6	1	1	0	1	9	1.8
*Journal of Trauma*	2	2	4	0	0	8	1.6
*Journal of Pediatric Orthopaedics*	3	5	0	0	0	8	1.6
*Lancet*	1	1	2	2	1	7	1.4
*Foot and Ankle Clinics*	3	0	1	2	0	6	1.2
*JAMA*	0	3	2	1	0	6	1.2
*Orthopaedics*	1	2	1	1	1	6	1.2
*Journal of Bone and Mineral Research: the Official Journal of the American Society for Bone and Mineral Research*	0	2	2	0	1	5	1.0
Total no. of references	87	124	108	106	69	494	
References per question	2.29	2.58	2.63	2.36	2.16	2.42	

**Table 4:** Reference Publication Year Relative to Orthopaedic In-Training Examination Year

Factor	2017	2018	2019	2020	2021	Total	Percentage of References
Year before and year of OITE	16	25	20	16	14	91	18.4
Within 5 yr	44	59	55	40	33	231	46.8
Within 10 yr	64	86	81	72	53	356	72.1
Within 15 yr	78	103	94	83	60	418	84.6
Total references	87	124	108	106	69	494	

On average, the most common taxonomic classification was T1 (46.1%), followed by T3 (37.7%) and T2 (16.2%). Table 5 demonstrates the distribution of questions in each taxonomic classification.

**Table 5:** Taxonomic Classification of Trauma Questions

Taxonomy	2017	2018	2019	2020	2021	Total	Percentage of Questions
T1	21	24	17	19	13	94	46.1
T2	5	5	6	13	4	33	16.2
T3	12	19	18	13	15	77	37.7
Total questions	38	48	41	45	32	204	

## Discussion

This study provides an updated analysis of the OITE musculoskeletal trauma section to better inform both residents as they prepare for the annual examination and program directors as they develop their program's educational curriculum. Trauma questions continue to account for a substantial proportion of the examination, representing 16.6% of questions between 2017 and 2021. This is slightly more than the 15% that the AAOS aims for trauma to represent,^3^ but no difference was found compared with prior studies of the OITE that analyzed only questions defined by the AAOS as trauma questions between 2005 and 2009^9,10^ (*P* = 0.240). These studies reported that trauma questions accounted for between 18.3% and 19.3% of questions on the examination each year,^10^ averaging 18.8% of the examination between 2005 and 2009.^9^ By contrast, another study on 2004 to 2008 OITE questions reported that 28.3% of OITE questions were trauma-related questions,^8^ which is significantly more than that found in our study (*P* < 0.001). This difference is likely a reflection of different inclusion and exclusion criteria for trauma questions across studies. Many questions related to traumatic injuries can be categorized as both a trauma question and a question in another orthopaedic subspecialty. For example, a traumatic ankle fracture could be categorized as both a foot and ankle and a trauma question. In our study, we opted to exclude questions related to hand and spine trauma because these injuries are typically managed by their respective subspecialist as opposed to a general orthopaedic surgeon.

Imaging studies, including plain radiographs, CT, and magnetic resonance imaging, are important in diagnostic and treatment decisions in orthopaedics. This study found that images were included in nearly half of all trauma questions tested. In comparison, prior studies from over a decade ago reported that images were included in only 28.3%^9^ and 30.2%^10^ of questions. This represents a significant increase in trauma questions incorporating imaging in the past decade (*P* value <0.001). The authors hypothesize that the increased incorporation of images may reflect the transition from a paper-based examination to a computer-based examination in 2009,^9^ which allows the inclusion of higher quality imaging studies and makes it both easier and less expensive to include images. Of the questions containing images in our study, 87.0% contained radiographs either alone or in combination with other imaging modalities. By contrast, 95% of questions with images contained plain radiographs between 2005 and 2009.^10^ Residents preparing for the OITE should aim to become proficient in the interpretation of both advanced imaging modalities and plain radiographs, but given the clear emphasis on plain radiographic imaging on the examination, test takers should be aware that plain radiographs are still the most frequently tested.

OITE question writers are encouraged to cite 2 to 6 high-impact journals for each examination question.^2^ Le et al^2^ highlighted that the AAOS recommends references should be published within the past 10 years, which held true for 72.1% of all references cited in our study. The majority (94.9%) of references were primary literature sources, an increase from the 75% that was reported in a prior study of trauma questions.^8^ The three most frequently cited sources in our study, the *Journal of Orthopaedic Trauma, Journal of the American Academy of Orthopaedic Surgeons*, and *Journal of Bone and Joint Surgery, American Volume*, were also among the most frequently cited sources identified in the prior reviews of trauma OITE questions.^8-10^ Interestingly, only 6% of residents report using journals as their primary study resource, but journals were also rated by the same group of surveyed residents as the most useful for preparation for the OITE.^7^ In contrast to reading journals, Theismann et al^7^ report that most residents use Orthobullets (53%), ResStudy (26%), JBJS Clinical Classroom (3%), textbooks (8%), or other resources (4%) in preparation for the OITE. The preference toward utilization of online learning platforms for OITE preparation is supported by program directors, 85% of which recommend the use of Orthobullets and 65.7% recommend the use of ResStudy.^19^ This highlights that residents should be aware of consistently high-yield journals such as the *Journal of Orthopaedic Trauma*, *Journal of the American Academy of Orthopaedic Surgeons*, and *Journal of Bone and Joint Surgery, American Volume* to supplement the secondary references they often use to prepare for the OITE.

As first described by Buckwalter et al^18^ in 1981, the OITE aims to test the complex cognitive processes necessary for the practice of orthopaedics. Questions are thus designed to fall into one of three different taxonomic classifications based on the cognitive processes required to correctly answer them. Taxonomy 1 questions test recognition and recall of information, taxonomy 2 questions test comprehension and interpretation of information, and taxonomy 3 questions test problem solving.^2,18^ Our study found that slightly less than half of questions (46.1%) were T1 questions, which represents a slight decrease from the prior analysis of AAOS-defined trauma questions that reported 60.6%^9^ and 69.0%^10^ of questions to be T1 (*P* < 0.001). The next most frequently tested questions are T3 questions that represent 38% of trauma questions, increased compared with 19.7%^9^ and 21.6%^10^ on prior analysis of AAOS-defined trauma questions (*P* < 0.001). Together, these results suggest that although nearly half of questions still test recognition and recall, there is a slight shift toward inclusion of questions requiring higher levels of cognitive reasoning to correctly answer in our study relative to prior studies of AAOS-defined trauma questions. Residents can focus on case-based practice questions, which are more likely to require higher-order cognitive processing, to adequately prepare for the increased proportion of these questions.^20^ Of note, there was no difference in the taxonomic distribution of questions that our study reported relative to Seybold et al^8^'s analysis of 2004 to 2008 musculoskeletal trauma OITE questions (*P* = 0.18), which reported 50.8% T1, 11.0% T2, and 38.2% T3 questions. This study by Seybold et al included both AAOS-defined trauma questions and questions defined by AAOS under different domains, which the authors believed also tested musculoskeletal trauma concepts. The difference in inclusion criteria for this study limits our ability to directly compare it with our findings.

There are several limitations to our study. The trauma domain is broad and overlaps with nearly all 10 other domains on the OITE. In the design of this study, the authors opted to define trauma questions based on injuries typically treated by a general trauma-focused orthopaedic surgeon and thus excluded questions that could be considered traumatic spine injuries and traumatic hand injuries. Although the authors believe that this definition most accurately reflects the intended domain of question writers, the AAOS does not publish their definition of the musculoskeletal trauma domain for comparison nor do prior studies of the OITE trauma questions.^8-10^ The inability to directly compare the inclusion and exclusion criteria across studies limits our ability to compare our study findings with those reported in previous studies. As the AAOS stopped publishing the complete study guide with the official categorization of all questions in 2016,^14^ we are unable to compare the questions we categorized under the domain of trauma with those that the AAOS intended to test trauma principles. In addition, our study examines the five most recent OITE examinations that are available on AAOS's ResStudy. Greater understanding of trends in questions may have been possible if we were able to examine all questions since the most recent trauma question analysis in 2009.

This study highlights that trauma continues to account for a notable proportion of the OITE, which has increased somewhat in recent years. The development of a digital examination has resulted in a substantial increase in the use of images for the trauma questions. Consistent with prior reports, high-impact journals remain notable sources of information for the chosen questions, and questions emphasize higher-level cognitive processes (comprehension, interpretation, and problem solving compared with recognition and recall). The results suggest that strategies for residents should include interpretation of images, development of higher-level cognitive processes through case-based practice questions, and awareness of high-impact journals.
